# Co-regulation between young deaf and hard-of-hearing children and their caregivers: study protocol of *Calming Together*

**DOI:** 10.1186/s40359-025-03379-4

**Published:** 2025-09-24

**Authors:** Nikki Smit, Amy Szarkowski, Hedwig J.A. van Bakel, Anat Zaidman-Zait, Evelien Dirks

**Affiliations:** 1 Dutch Foundation for the Deaf and Hard-of-Hearing Child (NSDSK), Amsterdam, the Netherlands; 2 University of Massachusetts Boston, Boston, United States of America; 3 Tranzo, Tilburg University, Tilburg, the Netherlands; 4 Open University in the Netherlands, Heerlen, the Netherlands; 5 Tel Aviv University, Tel Aviv, Israel

**Keywords:** Deaf, Hard-of-Hearing, Co-regulation, Self-regulation, Executive function, Temperament, Sleep

## Abstract

**Background:**

Children who are deaf and hard-of-hearing (DHH) often experience more difficulties with self-regulation than their typical hearing peers. Self-regulation develops partly through internalization of regulation strategies provided by their caregivers during co-regulation. Co-regulation is a dynamic process through which caregivers and children influence and regulate each other’s emotions and physiological responses. Given the essential role of co-regulation in the development of self-regulation it is important to investigate co-regulation in DHH children and their caregivers, given the limited research that has examined this subject. The present paper describes a study, *Calming Together*, which aims to examine co-regulation in 2- and 3-year-old DHH children and their parents, investigating how different facets of child and parent self-regulation such as temperament, executive function (EF), general self-regulation, and sleep are related and how they contribute to co-regulation.

**Methods:**

*Calming Together* will be a longitudinal study with data collected at three points in time. Participants will include 2- and 3-year-old children with permanent bilateral hearing loss, along with their parents. Measures at Time 1 will include (a) child EF assessment, (b) an observation of parent-child interaction, and (c) parental questionnaires. Parents will also receive equipment and instructions so that they can complete an assessment of sleep using actigraphy and a sleep diary. At Time 2 and Time 3, completed three and six months after the home visit, parents will complete questionnaires on self-regulation and co-regulation.

**Discussion:**

The *Calming Together* study will be among the first to investigate co-regulation in young DHH children and their parents in ecologically valid home settings. By assessing both parent and child factors, it will provide insights into how co-regulation unfolds in daily life and how it relates to broader aspects of self-regulation. The study will also include both mothers and fathers to investigate their potentially unique contributions toward co-regulation and the development of self-regulation in DHH children. Findings may inform early intervention strategies to support parent-child interactions and enhance self-regulation in DHH children. The study was retroactively registered in Open Science Framework (OSF) on April 15th 2025 (10.17605/OSF.IO/CHEKA).

## Background

The image of a toddler having a temper tantrum after their parents told them “No” is an image familiar to many. One reason young children sometimes react this way is because their abilities to regulate their emotions and behaviors have not fully developed. *Self-regulation* enables us to monitor and modulate cognition, emotions, and behavior to achieve goals and adapt to changing circumstances [1]. Self-regulation is crucial, as it helps us to behave appropriately in social contexts and to achieve our goals. Unsurprisingly, better self-regulation is associated with improved social skills, better mental health, and enhanced academic performance later in life [2, 3].

The development of self-regulation begins in infancy and is considered one of the main developmental tasks during toddlerhood [4]. For some children, the development of efficient self-regulation is more challenging. Children who are deaf and hard of hearing (DHH) often experience more difficulties with self-regulation than their typically hearing peers [5–7]. A common explanation for self-regulation challenges among DHH children is lower language abilities in DHH children [8–11], as language abilities play a crucial role in the development of self-regulation. Another factor that may be associated with the development of self-regulation is co-regulation.

Children’s self-regulation in the first years of life develops partly through internalization of regulation strategies provided by their caregivers during co-regulation [12, 13]. *Co-regulation* can be defined as “a bidirectional linkage of oscillating emotional channels (subjective experience, expressive behavior, and autonomic physiology) between partners, which contributes to emotional and physiological stability for both partners in a close relationship” [14, p. 203]]. In other words, co-regulation is a dynamic process through which a caregiver and a child mutually influence and regulate each other’s emotions and physiological responses.

To date, co-regulation in DHH children and their caregivers has not been thoroughly studied. Most studies on parent-child interaction among DHH children and their caregivers have focused on joint attention, parental language input, and parental sensitivity [15]. These aspects of parent-child interaction are sometimes challenging for DHH children and their parents [16, 17]. Given the essential role of co-regulation in the development of self-regulation, it is important to investigate co-regulation in DHH children and their caregivers.

Parents of DHH children may need to adopt different approaches during parent-child interactions to meet their children’s needs and enhance co-regulation processes compared to parents of typically hearing children. For instance, parents’ use of their voice for soothing might be less accessible and effective for DHH children. However, it remains unclear which strategies parents of DHH children employ to calm their children in everyday situations and which strategies are most effective for addressing their children’s needs.

When examining co-regulation, it is important to consider both child and parent factors. For example, parents’ self-regulation ability is one factor that is known to be related to parent-child interaction [18] and may also impact co-regulation. Parents who experience difficulties with self-regulation are less likely to respond effectively to their child’s challenging behaviors [19]. In parallel, children with a highly reactive temperament who often experience intense negative emotions require more support in navigating and calming these emotions. Highly reactive children benefit most from interactions that promote the development of self-regulation skills, such as co-regulating with their parents. On the contrary, parents’ negative reactions to their children’s emotions are more likely to hinder the development of self-regulation skills [20]. Therefore, temperament is another factor to consider.

Executive functions (e.g., inhibitory control, working memory, cognitive flexibility) are likely related to co-regulation. Executive functions are cognitive processes needed to achieve effective self-regulation, as they enable us to organize and process information, plan and solve problems, as well as guide thoughts, emotions, and actions in goal-directed behavior [21]. As executive functions are a part of self-regulation, it is possible that they are also related to co-regulation. For example, one study found that mothers with poorer working memory performance were more likely to react negatively to children’s challenging behaviors [22].

Sleep has also been related to self-regulation and therefore may also be related to co-regulation. Research found that poor quality sleep has been associated with lower self-regulation in children both in the short and long term [23–25]. Similarly, relationships between sleep and self-regulation have been found in adults [26, 27]. Palmer and Alfano [27, p14] even suggest that sleep helps us to identify emotions in need of regulation, to select an appropriate regulation strategy, and to use it effectively. This mechanism may also apply to co-regulating with a child. For example, recognizing a child’s frustration and assisting them in calming down by acknowledging their emotion and soothing them.

To our knowledge, researchers have not yet studied the relationships between multiple aspects of parent and child self-regulation and co-regulation within a single study. Studying multiple aspects of self-regulation at once might provide insight into facets of self-regulation, such as temperament and executive functioning, which may also influence or be associated with co-regulation. Moreover, studies on parent-child interaction in children who are DHH have mostly focused on child characteristics, or more commonly, on parent characteristics such as educational level [16, 28]. In addition, most studies on the role of parent-child interaction in DHH children focused on mothers [29], despite evidence that fathers contribute uniquely to the development of children’s regulation skills [30]. For example, fathers tend to engage in greater levels of rough and tumble play, which demands increased regulatory efforts from both the parent and child [31].

The current study, *Calming Together*, aims to examine the process of co-regulation among DHH children and their parents. Specifically, it will investigate how co-regulation, parent self-regulation, and child self-regulation are related as well as how those factors contribute to the overall co-regulation process. *Calming Together* will incorporate measures that assess different aspects of self-regulation: self-regulation in general, temperament, executive function, and sleep.

Studies on co-regulation are often conducted in controlled environments, such as laboratories or research centers [32]. Although these studies have been crucial to advancing our understanding of co-regulation, it is important to study how co-regulation unfolds in everyday life. Therefore, *Calming Together* will study co-regulation in DHH children and their parents in daily routines (e.g., playing, cleaning-up, eating, sleeping) to enhance the ecological validity of the study as much as possible.

## Methods

### Aims of the study

1. To describe the process of co-regulation among young deaf and hard of hearing (DHH) children and their parents.
2. To determine how self-regulation and co-regulation are related in DHH children and their parents.

### Study design

*Calming Together* will be a longitudinal study, with data collected across three points in time, to examine co-regulation among parents and their 2- and 3-year-old DHH children (for an overview, see Table 1). The study will use a multi-method approach. Time 1 will be conducted in the homes of the families and will include EF tasks with the child and an observation of parent-child interaction. If both parents participate, an additional home visit will be conducted to observe parent-child interaction with the second parent. Additionally, parents will be asked to complete questionnaires on both parent and child characteristics, including self-regulation, EF, temperament, as well as questionnaires on co-regulation, and their child’s sleep and mealtime behaviors. During the initial home visit, parents will also receive equipment and instructions so that they can complete an assessment of sleep using actigraphy and a sleep diary for 7 days for both parents and the child.

**Table 1:** Timeline of data collection

Time 1	Time 2^a^	Time 3^b^
**Questionnaires** Background information Parent & child factors Self-regulation & co-regulation	**Questionnaire** Self-regulation & co-regulation	**Questionnaire** Self-regulation & co-regulation
**In-home Data Collection** Executive function child Parent-child interaction		
**Parent collected data** Sleep quality		

***Note***. ^a^3 months after Time 1 ^b^6 months after Time 1

At Time 2 and Time 3, completed at 3- and 6-months after Time 1, parents will complete additional questionnaires on parent and child self-regulation, and co-regulation. Data collection started at the beginning of 2024 and will end in the summer of 2025.

### Participants

The study will include children with permanent bilateral hearing loss, 2- and 3-years of age, enrolled in family-centered early intervention (FCEI) in the Netherlands. Children with additional difficulties (e.g., medical complexities or disabilities) will be included if they are able to sit and engage with their parents. Children with bilateral hearing loss - ranging from mild to profound levels of hearing loss – whether they use no hearing equipment, hearing aids, bone-anchored hearing aids (BAHA)), or cochlear implants (CIs), will be included in the present study. Children from multilingual families will also be allowed to participate, if the parents are able to communicate in Dutch, Dutch Sign Language, or English. To the extent possible, the study will include two parents of the child, inclusive of fathers, mothers, or same-sex parents. Both typical hearing and DHH parents will be welcomed.

### Inclusion criteria

1. Children with permanent bilateral hearing loss ages 2- and 3- years of age.
2. Children who are deaf with additional challenges (medical complexities or additional disabilities) will be included.

### Exclusion criteria

1. Children who are unable to engage with their parents.
2. Children with visual impairments.
3. Parents who are not able to communicate in Dutch, Dutch Sign Language, or English.

An a priori power analysis was conducted using G*Power (version 3.1.97) to determine the minimum number of participants needed to test the study hypotheses. To assess the relations between the different facets of co-regulation using correlations, we will need to include *N* = 50 participants to achieve 80% power for detecting a moderate to large effect, at a significance criterion of α = 0.05. As this study is among the first to investigate co-regulation in this way, it was not possible to base the effect size on prior studies. Therefore, a medium to large effect size was used to calculate the sample size.

Participants will be recruited from FCEI centers of the Dutch Foundation for the Deaf and Hard-of-Hearing Child (NSDSK) and Pento. Participants will also be recruited through social media posts about the study. This study has been reviewed and received ethical approval from the Ethics review Board from Tilburg University (TSB_RP749). The expected date of completion of participant recruitment is July 31st 2025.

### Data collection

An overview of the study design can be found in Table 2. During the home visit at Time 1, the experimenter will conduct three tasks with the child. All three tasks will be video recorded and will be conducted while sitting at a table, either in a highchair or on their parents’ lap. Afterward, parent-child interaction (PCI) during five consecutive tasks will be video recorded: free play, building a block tower, a frustration task, an additional free play session, and a clean-up task. If two parents participate, another home visit will be scheduled approximately one week after the initial home visit to record the parent-child interaction sequence with the other parent. The parent-child interactions of the two parents will be recorded on separate occasions to minimize the burden on the child and help ensure that the toys provided remain interesting to the child. During the recording of parent-child interaction with the second parent, the frustration task will be omitted, as it will be familiar to the child.

In addition, parents and their DHH child will be asked to wear an actigraphy watch during sleep for a period of seven days to measure sleep quality. Parents will also be asked to fill in a sleep diary to aid in the actigraphy analysis. Additionally, parents will complete online questionnaires on both parent- and child EF, self-regulation, temperament, co-regulation, and child mealtime behavior and sleep. The link to the questionnaire will be sent via email.

**Table 2:** Overview of the study design of calming together including the constructs and instruments

In-home assessment	Construct	Instrument
EF child	Inhibitory control	Baby Stroop [33]
	Working memory	Memory Span Spin the Pots [34]
	Effortful control	Gift Delay [35]
Parent-child observation	Co-regulation	Dyadic Interaction Coding [36]
Sleep parent & child	Sleep quality	Actigraphy & sleep diary
**Questionnaires**	**Construct**	**Instrument**
Child measures	Executive function	Behavior Rating Inventory of Executive Function Preschool (BRIEF-P) [37]
	Temperament	Early Child Behavior Questionnaire (ECBQ) – Very Short [38]
	Self-regulation	Infant Toddler Symptom Checklist (ITSC) [39]*
	Sleeping behavior	Brief Infant Sleep Questionnaire (BISQ) – Revised [40]
	Mealtime behavior	Mealtime Behavior Questionnaire (MBQ) [41]
	Language	Schlichting [42]
Parent measures	Executive function	Behavior Rating Inventory Executive Function Adult (BRIEF-A) [43]
	Temperament	Adult Temperament Questionnaire (ATQ) [44]
	Self-regulation	Emotion Regulation Questionnaire (ERQ) [45]*
Parent-child measures	Co-regulation	Coping with Toddlers’ Negative Emotions Scale (CTNES) [46]*
		Co-regulation Strategies Questionnaire [47]*

Note. * These instruments will also be used at time 2 and 3

At three months and six months following the final home visit, parents will be asked to complete questionnaires regarding parent and child self-regulation, as well as co-regulation strategies. These questionnaires make up Time 2 and Time 3.

### Child measures

#### Executive function

To measure executive function, a combination of behavioral and parent-reported measures will be used. The behavioral measure will consist of three separate tasks, all targeting different aspects of executive function (i.e., inhibitory control, working memory, effortful control). The tasks will be conducted in a set order, as described below. The conditions of the Baby Stroop and Memory Span Spin the Pots tasks will be counterbalanced to reduce bias.

**Inhibitory control**. The Baby Stroop task [33] will be used to measure inhibitory control using a baby-sized cup and spoon alongside a regular-sized cup and spoon. The child will be taught that the parent uses the big cup/spoon, and the baby uses the small cup/spoon. After confirming that the child understands the rule, the experimenter will switch the rule and teach the child that the baby uses the big cup/spoon, and the parent uses the small cup/spoon. The experimenter will then ask the child six times in a set order who uses the cup/spoon. The total score is the number of correct trials for both cup and spoon trials.

**Working memory**. A modified version of the Memory Span Spin the Pots task (MMSP) [34] will be used to assess working memory. This task will involve two conditions: “no cover and no rotation” (nCnR) and “cover and rotation” (CR). In the nCnR condition, a spinning platter with upside-down cups will be placed in front of the child. The experimenter will hide a toy under one of the cups, and after three seconds, the child will be asked to retrieve it. In the CR condition, the experimenter will cover the cups with cloth, rotate the platter a quarter turn clockwise, and then ask the child to find the toy.

Each condition will begin with a practice trial followed by three levels of increasing difficulty. The levels, with two trials each, will progress from hiding one toy under three cups to hiding three toys under seven cups. The child will be allowed one extra attempt to retrieve all toys during each trial. If the child is unable to retrieve all the toys in both trials of a level, even after the extra attempt, the task will be discontinued. The total score will be calculated by summing the scores for each level and correcting for chance.

**Effortful control**. Effortful control will be assessed with a delay of gratification task involving a gift [35]. The experimenter will present a gift bag to the child and inform the child that they need to leave the room (for three minutes) to get the bow, instructing the child to not touch the bag until the experimenter returns. During this time, the parent will remain present but will not interact with the child.

The child’s behavior will be coded for actions such as touching, opening, or looking into the bag, placing a hand inside, removing the gift, or leaving the chair. The time between when the child is informed of the need to wait for the experimenter to return to when the child acts will be recorded as a latency score. Latency scores will range from 0 (child acts immediately) to 180 (child never performs the action). The total score will be the mean of these latencies.

**Parent-reported executive function**. The Dutch version of the Behavior Rating Inventory of Executive Function – Preschool (BRIEF-P) [36] will be used as a parent-report measure of child executive function. The BRIEF-P includes 63 statements about child behavior. Parents will rate how often their child has exhibited each behavior in the past six months on a 3-point Likert scale ranging from 1 (never) to 3 (often). Higher scores indicate more executive function difficulties.

#### Temperament

The Dutch version of the Early Childhood Behavior Questionnaire (ECBQ) – Very Short [37] will be used to measure child temperament. The ECBQ – Very Short consists of 36 items describing child behaviors. Parents will rate how often their child exhibited each behavior in the past two weeks on a 7-point Likert scale, from 1 (never) to 7 (always), with an option to select “not applicable” if the situation never occurred.

#### Self-regulation

The Self-Regulation subscale of the Dutch version of the Infant Toddler Symptom Checklist (ITSC) [38] will be used to measure child self-regulation. The ITSC includes nine statements about child characteristics. Parents will rate their agreement with each statement on a 7-point Likert scale ranging from 1 (strongly disagree) to 7 (strongly agree). Higher scores indicate better self-regulation ability.

#### Sleep

The Brief Infant Sleep Questionnaire – Revised (BISQ-R) [39] will be used to assess child sleep behavior. The BISQ-R consists of 34 items addressing sleep patterns, bedtime routines, and sleep problems. The scores will be anonymized and sent to the team that developed the BISQ-R to calculate the total scores [39].

#### Mealtime behavior

The Mealtime Behavior Questionnaire (MBQ) [40] will be used to measure child behavior during mealtime. The MBQ consists of 31 items assessing behaviors. Parents will rate how often their child exhibited these behaviors during mealtime on a 5-point Likert scale, from 1 (never) to 5 (always). A higher score indicates more mealtime behavior problems.

#### Spoken language

The subtests Expressive Vocabulary (EV) and Expressive Grammar (EG) from the Schlichting Test for Language Production-II [41] will be used to assess spoken language ability. The Schlichting was developed and standardized for children between 2 and 7 years old. Word quotient and sentence quotient scores will be determined using a representative sample from the Netherlands (aged 2–7 years). These measures are already available on record for all children, as they are part of the assessment protocol used by the FCEI organizations participating in this study.

### Parent measures

#### Executive function

The Behavior Rating Inventory of Executive Function – Adult (BRIEF-A) [42] will be used to assess parent executive function. The BRIEF-A consists of 75 statements on behavior. Parents will report how often they exhibited these behaviors in the past month on a 3-point Likert scale from 1 (never) to 3 (often). Higher scores indicate more executive function difficulties.

#### Temperament

The Adult Temperament Questionnaire (ATQ) short form [43] will be used to assess parent temperament. The ATQ short form includes 77 statements on characteristics. Parents will rate the extent that the statements apply to them on a 7-point Likert scale, from 1 (not at all applicable) to 7 (completely applicable), or indicate if they have never been in that situation.

#### Self-regulation

The Emotion Regulation Questionnaire (ERQ) [44] will be used to measure parent self-regulation. The ERQ includes 10 statements about emotions. Parents will rate their agreement with each statement on a 7-point Likert scale, from 1 (totally disagree) to 7 (totally agree). The item scores will be summed into two separate scales reflecting two types of strategies: cognitive reappraisal and expressive suppression. High scores on the scales reflect frequent use of the respective strategies.

### Co-regulation

#### Self-reported co-regulation strategies

**CTNES**. The Coping with Toddlers’ Negative Emotions Scale (CTNES) [45, 46] will be used to measure parent self-reported use of co-regulation strategies. The CTNES presents 12 hypothetical situations with seven possible strategies. Parents will rate the likelihood of using these strategies on a 3-point Likert scale, from 1 (very unlikely) to 3 (very likely). Item score will be summed into two separate scales reflecting two types of strategies: supportive and unsupportive strategies. Higher scores on the scales suggest frequent use of the respective strategies.

**Co-regulation Strategies Questionnaire**. The Co-regulation Strategies Questionnaire [47] will also be used to measure self-reported co-regulation strategies. This questionnaire includes six emotion regulation strategies. Parents will rate how often they use each of these strategies on a 6-point Likert scale, from 1 (never) to 6 (always).

#### Observation of co-regulation

Co-regulation will be investigated by observing parent-child interaction during five consecutive tasks for a total of 15 min. The five tasks consist of free play (3 min), building a block tower (3 min), a frustration task (4 min), additional free play (3 min), and a clean-up task (3 min). If more than one parent participates, a second recording will be made of parent-child interaction with the other parent(s) (which will be administered during a separate session), and the frustration task will be omitted.

The five assessment tasks will be conducted in the home of the family. Parents will be given instructions on the separate tasks in advance of the interaction. During free play, parents will play with their child as they normally would, with age-appropriate toys provided on a playmat. In the block tower episode, parents and children will build a tower using wooden blocks. The frustration episode is adapted from the attractive toy in a transparent box procedure from the Laboratory Temperament Battery (Lab-TAB) [48]. The parent and child will be given an attractive toy in a clear locked box with a set of keys, none of which fit the padlock. Parents will be instructed that unlocking the box is the child’s responsibility and should refrain from offering or providing physical assistance. After 4 min, the experimenter will provide the correct key. After unlocking the padlock, the parent and child will play as usual for another 3 min to recover from the frustration episode. Finally, the parent will instruct the child to clean up the toys; while the parent is allowed to help, the clean up task will be the child’s responsibility.

Parent-child interactions will be coded using the Lunkenheimer Dyadic Coding System [49], and the codes will be recorded in the Mangold INTERACT program [50]. Parent and child affect will be coded separately on the following scale: negative, neutral, low positive, and medium/high positive. Affect will be micro-coded on a frame-by-frame basis.

Parent and child behaviors will also be coded. Parent behavior coding will include proactive structure, positive reinforcement, emotional support, teaching, directive, engagement, disengagement, intrusion, and negative discipline. Child behavior that will be coded include compliance, persistence, social conversation, solitary/parallel play, noncompliance, disengagement, and emotion dysregulation. Behavior will be micro-coded on a second-by-second basis.

### Sleep quality

The sleep quality of both parents and children will be measured for 7 consecutive days using actigraphs (Motionlogger Micro Watches, Ambulatory Monitoring, Inc., Ardsley, NY) in zero crossing mode worn during sleep. Actigraphs are wristwatch-like devices that register movement and are non-invasive. Parents will also be asked to complete a sleep diary to aid in determining the periods of sleep. Actigraphy data will be scored with Action-W^®^ software using empirically derived algorithms. The parameters that will be derived from the data will be sleep duration, number of awakening episodes, and sleep efficiency which is an index for sleep quality and is calculated by the dividing the time spent asleep by the total sleep period.

### Data management

At the start of the home visit, informed consent will be obtained. Data will not be shared with third parties and will be anonymously stored in a safe location. Only the first author and principal investigator will have access to all data collected during the study. Additional researchers involved with coding will have access only to the relevant and anonymized data. Articles published on the data will only include processed data and will be fully anonymized.

## Discussion

*Calming Together* is among the first research investigating co-regulation in DHH children and their parents. Findings of *Calming Together* will provide insight into the process of co-regulation between DHH children and their parents, providing valuable insights into how DHH children and their parents influence each other’s affect during interactions and which strategies parents use during co-regulation with their children during daily situations. These findings are relevant to theory and practice for multiple reasons.

Firstly, *Calming Together* will study co-regulation in the homes of families, during daily situations and routines. This study will explore co-regulation in as ecologically valid manner as possible; thus, the findings will more likely be representative of actual co-regulation between DHH children and their parents. Secondly, *Calming Together* will intentionally include both mothers and fathers, making it possible to investigate potentially unique contributions of all parents during co-regulation and the development of self-regulation in DHH children. Thirdly, the present study will assess both parent- and child-factors related to co-regulation. The research team will investigate the relationships between these parent- and child-factors and co-regulation to gain understanding into the factors related to co-regulation. Specifically, *Calming Together* will offer insight into different aspects of self-regulation, such as executive function, temperament, and sleep in both parents and children. Mealtime behaviors children and their relation with co-regulation will also be explored. Lastly, findings obtained through the *Calming Together* project are likely to be used to develop interventions aimed at improving parent-child co-regulation, which can contribute to improvement of the self-regulation development of DHH children. The findings can also be used to inform professionals in FCEI about how to best help parents to support their DHH child’s self-regulation development.

The results of *Calming Together* will be made publicly available to researchers, healthcare professionals working in FCEI in the Netherlands, as well as parents and caregivers of DHH children. The findings of *Calming Together* will be shared with researchers and healthcare professionals as presentations at both at national and international conferences and as articles in peer-reviewed scientific journals. Parents and caregivers of DHH children will be informed about study findings through visual graphics and factsheets.

## Data Availability

No datasets were generated or analysed during the current study.

## Abbreviations

DHH: Deaf and Hard-of-Hearing
CI: Cochlear Implant
MMSP: Memory Span Spin the Pots
nCnR: No Cover and no Rotation
CR: Cover and Rotation
BRIEF: Behavior Rating Inventory of Executive Function
ECBQ: Early Child Behavior Questionnaire
ITSC: Infant Toddler Symptom Checklist
BISQ: Brief Infant Sleep Questionnaire
MBQ: Mealtime Behavior Questionnaire
EV: Expressive Vocabulary
EG: Expressive Grammar
ATQ: Adult Temperament Questionnaire
ERQ: Emotion Regulation Questionnaire
CTNES: Coping with Toddlers’ Negative Emotions Scale
